# Thinner changes of the retinal nerve fiber layer in patients with mild cognitive impairment and Alzheimer’s disease

**DOI:** 10.1186/s12883-015-0268-6

**Published:** 2015-02-21

**Authors:** Dachuan Liu, Lina Zhang, Zhen Li, Xuxiang Zhang, Yue Wu, Huiqing Yang, Baoquan Min, Xinqing Zhang, Daqing Ma, Yan Lu

**Affiliations:** Department of Ophthalmology, Xuan Wu Hospital, Capital Medical University, Beijing, 100053 China; Department of Ophthalmology, The First Affiliated Hospital of Zhengzhou University, Zhengzhou, 453003 China; Department of Neurology, Xuan Wu Hospital, Capital Medical University, Beijing, 100053 China; Anaesthetics, Pain Medicine and Intensive Care, Department of Surgery and Cancer, Imperial College London, Chelsea and Westminster Hospital, London, SW10 9NH UK

**Keywords:** Alzheimer’s disease, Mild cognitive impairment, Retinal nerve fiber layer, Optical coherence tomography

## Abstract

**Background:**

Alzheimer’s disease (AD) is the most common form of dementia and patients often have visual disorders. Mild cognitive impairment (MCI) is characterized by a memory deficit when compared with those of a similar age and education level which could indicate an earlier onset of AD. The aim of this study is to measure the changes of the retinal nerve fiber layer (RNFL) thickness of AD and MCI patients in comparison with the normal age controls.

**Methods:**

The RNFL thickness was assessed using optical coherence tomography (OCT) in patients with MCI, AD (mild, moderate and severe) and the age matched controls.

**Results:**

The thickness of RNFL in the superior quadrant and total mean values are gradually and significantly decreased from MCI to severe AD when compared to that in the controls. There is also a significant reduction of the retinal nerve fiber layer in the inferior quadrant in severe AD patients.

**Conclusions:**

Our data indicate that the retinal nerve fiber layer degeneration is paralleled with dementia progression. Owing to its non-invasive and cost effective nature, monitoring RNFL thickness may have a value in assessing disease progression and the efficacy of any treatments.

## Background

Alzheimer’s disease (AD) is the most common form of dementia and is characterized by cognitive deficits including progressive memory disturbances, aphasia, apraxia and agnosia. AD patients also have visual problems affecting visual acuity [1], stereopsis, colour vision, spatial contrast sensitivity and ocular motility [2]. The typical pathological changes in AD are senile plaques and neurofibrillary tangles in the central nervous system (CNS). Mild cognitive impairment (MCI) is defined as impairment in cognitive functions with otherwise normal performance of activities of daily life [3]. MCI is a broad term that encompasses several subtypes of cognitive dysfunction. Amnesic MCI patients may show an early transitional stage development of AD and have memory impairment, but without dementia [4]. 10% -15% MCI suffers are most likely to progress to AD per year while 1% to 2% of healthy people are in a risk developing to AD [5,6].

Optical coherence tomography (OCT) is a well-established non-invasive examination that can assess the thickness of retinal nerve fiber layer (RNFL) and is used in various ophthalmologic diseases including glaucoma, ocular hypertension, optic neuropathy and multiple sclerosis [7]. Previous reports have demonstrated a possible degeneration of the RNFL in AD [8-15]. For example, a postmortem study by Hilton et al., found widespread axonal degeneration in the optic nerves in 8 out of 10 AD sufferers [16]. Sadun’s work also suggested that the degeneration of ganglion cells were mainly observed in large M-cell axons [8]. However, other studies failed to support those findings [17,18], indicating that methodological differences may be responsible for those different findings but this warrants further study. The aim of the present study was to determine with a sophisticated OCT method whether the thickness of the RNFL is proportionally reduced from MCI patients, mild AD to severe AD patients compared to that in the age-matched healthy controls.

## Methods

After approval from the Ethics committee of Xuanwu Hospital, Capital Medical University, Beijing, China and written informed consent was obtained, 26 MCI, 24 mild AD patients, 24 moderate AD patients, 19 severe AD patients and 39 age-matched controls were enrolled into the study. All patients and controls were examined for visual acuity, refractive error, intraocular pressure (IOP), anterior and posterior segment biomicroscopy, dilated fundus examination.

All AD patients were diagnosed by the AD group of neurologists in the department of Neurology in Xuanwu Hospital according to the National Institute of Neurologic and Communicative Disorders and Stroke–Alzheimer’s Disease and Related Disorders Association (NINCDS-ADRDA) [19] and the Diagnostic and Statistical Manual of Mental Disorders (DSM IV) criteria [20].

Each of the MCI patients are diagnosed by three neurologists in the department of Neurology Xuanwu hospital according to Petersen criteria [21]. The criteria for controls were: (1) no memory complaints; (2) MMSE scores above 28. Additional criteria requirements for all study subjects are: (1) Diopters: spherical −3.00DS ~ +3.00DS, cylinder −3.00 DC ~ + 3.00 DC, anisometropia ≤ 2D;(2) IOP measured three times < 21 mmHg; Exclusion criteria was: glaucoma and increased intraocular pressure, retinal detachment, retinal artery occlusion, optic neuropathy, ocular trauma or surgery, diabetes mellitus, hypertension, cerebral infarction and other diseases which may affect RNFL thickness.

OCT evaluations were done according to the standard procedures using a STRATUS OCT 3 (Carl Zeiss AG, Oberkochen, Germany). Near infrared super-luminescent diode light allows the OCT to generate two dimensional images of the retina. RNFL thickness was circularly measured around the papilla (optic disc: 3.4 mm) and repeated three times per quadrant (superior, inferior, nasal and temporal) and the average of the 12 values were used for each eye as expressed in μm. The threshold of scanning signal was set to be ≥ 6.

Data are reported as mean ± SD and statistical analysis was performed with SPSS 16.0 (SPSS Inc, Chicago, IL, USA). The differences about gender constituent ratio among controls, MCI, mild AD, moderate AD and severe AD were compared with chi-square test. The differences about age, IOP, and RNFL thickness among five groups were evaluated with one-way ANOVA followed by post hoc comparison with Bonferroni correction to test the data between groups. A *p* < 0.05 was considered to be of statistical significance.

## Results

There was no statistically significant difference in gender, age and IOP among five groups (*P* > 0.05) (Table 1).

**Table 1:** **The clinical data of patients with MCI, mild, moderate, severe AD and control groups (mean ± SD)**

		**MCI**	**MI AD**	**MO AD**	**SE AD**	**control**		***P*** **value**
		**(n = 26)**	**(n = 24)**	**(n = 24)**	**(n = 19)**	**(n = 39)**		
Sex	M	12	9	11	9	17	*X* ^2^ = 0.370	>0.05
	F	14	15	13	10	22		
Age (y)		70.2 ± 6.5	71.3 ± 4.9	70.8 ± 6.1	72.1 ± 4.6	69.7 ± 7.8	F = 1.341	>0.05
IOP (mmHg)		16.1 ± 1.3	15.9 ± 2.1	15.1 ± 1.2	14.4 ± 1.2	15.9 ± 0.9	F = 2.260	>0.05
VA (logMAR)		0.16 ± 0.08	0.21 ± 0.10	0.33 ± 0.01	0.35 ± 0.03	0.06 ± 0.09	F = 3.501	<0.05

MCI: Mild cognitive impairment; AD: Alzheimer’s disease; n: eyes; MI: mild; MO: moderate.

SE: severe; M: male; F: female; IOP: intraocular pressure; VA: visual acuity.

**Figure 1:**
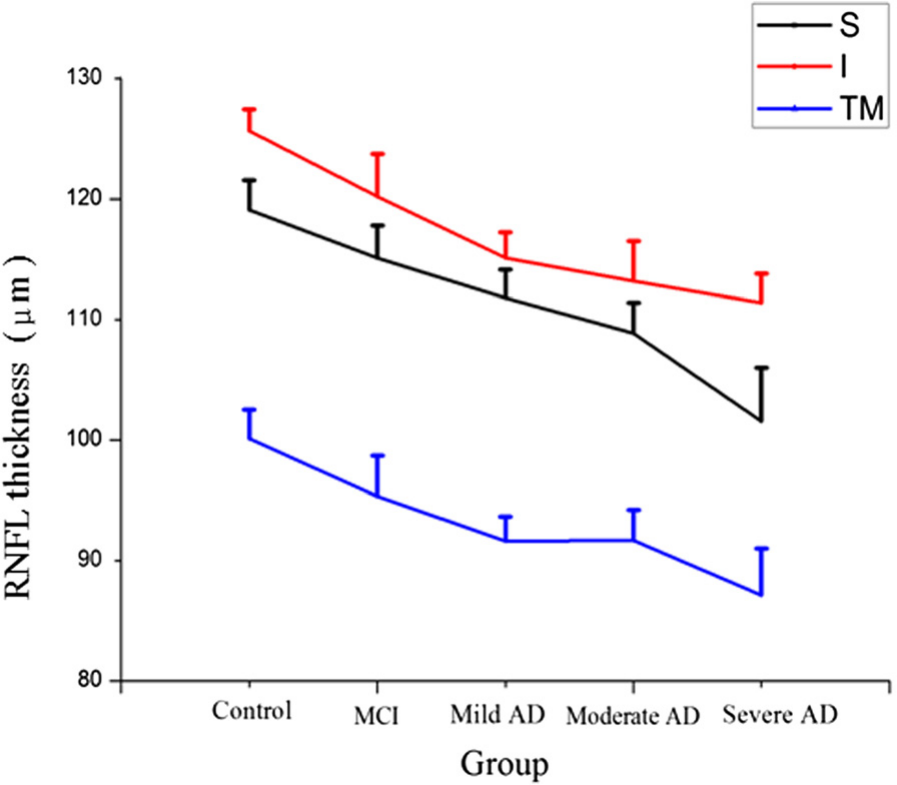
**RNFL thickness in patients with MCI, mild, moderate, severe AD and control subjects.** Line graph constructed with data from Table 2 represents the changing trend of mean RNFL thickness (μm) plus variations in the superior quadrant (s), inferior quadrant (I) and total mean (TM) along with the disease progression.

There was a significant difference between the normal control group and MCI and 134 mild AD, moderate AD or severe AD respectively in superior quadrant, inferior 135 quadrant and total mean RNFL thickness (*p* < 0.05) but no significant difference in nasal and temporal quadrant (*p* > 0.05). In the superior quadrant and total mean RNFL, compared with that in the normal control group, the RNFL thickness of the MCI, mild dementia, moderate dementia and severe dementia group were reduced 139 and there was a statistically significant difference (*p* < 0.05). Furthermore, when compared with the MCI group, the RNFL thicknesses of moderate and severe AD group were significantly decreased (*p* < 0.05). There was no significant difference in RNFL thickness among mild AD, moderate AD and severe AD groups. In the inferior quadrant, there are no significant differences (*p* > 0.05) in the MCI group, mild AD group and moderate AD group compared with the control group. In the total mean RNFL, compared with that in the normal control group, the RNFL thickness of MCI group, mild AD, moderate AD, severe AD were gradually reduced (*p* < 0.05) (Table 2). Furthermore, when these data were plotted together, it clearly revealed that there was a falling trend along with the disease progression (Figure 1).

**Table 2:** **RNFL thickness (μm) in patients with MCI, mild, moderate, severe AD and control subjects (mea ± SD)**

	**MCI**	**mild AD**	**moderate AD**	**severe AD**	**control** ***F*** **value**
S	115.14 ± 13.51^▲^	111.78 ± 11.67^▲^	108.89 ± 12.42^▲■^	101.56 ± 19.32^▲■^	119.10 ± 15.34
					2.012*
I	120.23 ± 18.04	115.11 ± 10.56	113.23 ± 16.21	111.41 ± 10.64^▲■^	125.67 ± 11.23
					5.549**
N	74.80 ± 12.36	69.76 ± 11.64	64.41 ± 15.87	61.12 ± 17.54	79.98 ± 12.87
					1.035
T	63.78 ± 13.16	61.17 ± 12.14	60.43 ± 11.87	60.41 ± 10.82	67.34 ± 15.27
					1.256
M	95.37 ± 17.11^▲^	91.61 ± 10.10^▲^	91.68 ± 12.37^▲■^	87.13 ± 17.05^▲▲■^	100.12 ± 15.01
					7.985**

S: superior of Peripapillary RNFL thickness; I: inferior of Peripapillary RNFL thickness; N: nasal of Peripapillary RNFL thickness; T: temporal of Peripapillary RNFL thickness; M: total mean of RNFL thickness; Compared with the control group:^▲^ *P* < 0.05,^▲▲^ *P* < 0.01; comparison to MCI:^■^ *P* < 0.05 F test: *p < 0.05, **p < 0.001.

## Discussion

Our data indicated the thickness of RNFL in the superior quadrant and total mean RNFL was gradually decreased that with the disease progression from MCI to severe AD. There was also a reduction of the retinal nerve fiber layer in inferior quadrant in severe AD patients.

In line with our data, a previous study measuring peripapillary RNFL in AD by OCT showed that a significant thinning change of the RNFL was found in the superior quadrant in patients with AD compared with control subjects. There were no significant differences in the inferior, temporal or nasal RNFL thicknesses between the groups [22]. Our previous study found that the superior and inferior quadrants nerve fiber layer thickness was thinning in the AD patients compared with the healthy controls measured by OCT [23]. Other reports [24,25] indicated that there is a clear reduction of RNFL thickness, involving all four quadrants in AD and MCI patients. Our current data suggest that there is the reduction in retinal nerve fiber layer in superior quadrant selectively in the early AD. Accompanying with the development of AD, the degeneration of the retinal nerve fiber layer appears not only in superior quadrant, but also in inferior quadrant. The reason for such selective thinning RNFL in the superior region remains unknown. Anatomically, axons from the superior retina project via the parietal lobe portion of the optic radiation to the cuneal gyrus of the primary visual cortex, whereas axons from the inferior retina project to the lingual gyrus. In a histopathology study of cortical damage in AD, Armstrong [26] found a greater density of senile plaques and neurofibrillary tangles in the cuneal gyrus than in the lingual gyrus, and suggested that this difference may explain the predominantly superior RNFL defects in AD.

Several previous reports indicate that visual problems in AD were attributed to nerve degeneration in the primary visual cortex [27-29]. However, there have been increasing evidence that the primary visual pathways (the optic nerve and retinal degeneration) may also contribute to the visual disorders. For example, there are publications suggesting that the optic nerve and retinal degeneration existed in patients with AD [5,7-9,16]. That is the case in our study reported here. Accompanying the development of AD, the degeneration of the retinal nerve fiber layer appears not only in superior quadrant, but also in inferior quadrant. However, some of the literature did not support the notion. For example, a previous study showed that the deficits in visual function that are known to occur in dementia of the Alzheimer’s type are not related to optic nerve head structural anomalies, at least in the earlier stages of the disease [30]. Kergoat et al. analyzed fundus images, captured by scanning laser polarimetry, and they found no differences in the RNFL thickness observed between AD and healthy subjects [30]. Hence, this warrants further study to identify the true reason of visual disorders in AD and also to clarify whether visual disorders are also due to disorders of visual cortex and/or primary visual pathways.

Amyloid-beta (Aβ) plaques which is also known as senile plaques have been reported in postmortem retinal tissue from patients with AD and the mouse model of AD [31-33]. We found the degree of the retinal nerve fiber layer degeneration deteriorated gradually along with AD development which indicates the retinal Ganglion Cell (RGC) losses may be correlated with the duration of AD progression [2]. Interestingly, amyloid protein has also been shown to be associated with the degeneration of RGC in a mouse model of glaucoma [34]. Therefore, amyloid accumulation in the retina of AD patients may result in degeneration of RGC in parallel to amyloid-related neurodegeneration in the cerebrum.

MCI constitutes a risk factor for AD. The RNFL thickness occurred in MCI patients being found in our study is striking although this warrants further study. If this is true, then it would be very valuable for patients to have general screening with OCT to further support diagnosis for a possibility to detect the earlier onset of MCI. Extensive research is being devoted to identifying individuals who are likely to develop AD from MCI [6]. Our data showing that RNFL thickness in MCI is reduced may be further evidence indicating MCI progression in patients while a follow-up study is needed to explore how the degree and duration of the RNFL relates to the progression from MCI to AD. However, cautions must be taken due to the following reasons: 1) This is a pure observational cohort study not a trial. Large sample size is needed to further verify our current findings; 2) Petersen criteria was used to define MCI without further biomarkers or even evidence from PET imaging; 3) A time not spectral domain OCT was used and hence lower resolution images can be problematic for data accuracy. Nevertheless, our data reported here may guide neurologists to consider OCT to be additional tool for better diagnosis and/or treatment monitoring of dementia patients although it is very plausible to be used to clarify the severity of dementia.

## Conclusions

OCT is a safe and non-invasive method which has been used to assess retinal degeneration in various ophthalmologic and neurological disorders. From our experience and the data reported here, it could be suggested that OCT can be used to improve MCI diagnosis in individuals clinically affected by subtle memory disturbances and to monitor the progression of Alzheimer’s disease and evaluate effectiveness of any treatments.

## Abbreviations

AD: Alzheimer’s disease
MCI: Mild cognitive impairment
RNFL: Retinal nerve fiber layer thickness
OCT: Optical coherence tomography
CNS: Central nervous system

## References

[1] Kesler A, Vakhapova V, Korczyn AD, Naftaliev E, Neudorfer M (2011). Retinal thickness in patients with mild cognitive impairment and Alzheimer’s disease. Clin Neurol Neurosurg.

[2] Risacher SL, Wudunn D, Pepin SM, MaGee TR, McDonald BC, Flashman LA (2013). Visual contrast sensitivity in Alzheimer’s disease, mild cognitive impairment, and older adults with cognitive complaints. Neurobiol Aging.

[3] Werner P, Korczyn AD (2008). Mild cognitive impairment: conceptual, assessment, ethical, and social issues. Clin Interv Aging.

[4] Petersen RC, Doody R, Kurz A, Mohs RC, Morris JC, Rabins PV (2001). Current concepts in mild cognitive impairment. Arch Neurol.

[5] Petersen RC, Stevens JC, Ganguli M, Tangalos EG, Cummings JL, DeKosky ST (2001). Practice parameter: early detection of dementia: mild cognitive impairment (an evidence-based review). Report of the quality standards subcommittee of the American Academy of Neurology. Neurology.

[6] Petersen RC (2004). Mild cognitive impairment as a diagnostic entity. J Intern Med.

[7] Jaffe GJ, Caprioli J (2004). Optical coherence tomography to detect and manage retinal disease and glaucoma. Am J Ophthalmol.

[8] Sadun AA, Bassi CJ (1990). Optic nerve damage in Alzheimer’s disease. Ophthalmology.

[9] Blanks JC, Torigoe Y, Hinton DR, Blanks RH (1996). Retinal pathology in Alzheimer’s disease I Ganglion cell loss in foveal/parafoveal retina. Neurobiol Aging.

[10] Valenti DA (2010). Alzheimer’s disease: visual system review. Optometry.

[11] Larrosa JM, Garcia-Martin E, Bambo MP, Pinilla J, Polo V, Otin S (2014). Potential new diagnostic tool for Alzheimer’s disease using a linear discriminant function for fourier domain optical coherence tomography. Invest Ophthalmol Vis Sci.

[12] Polo V, Garcia-Martin E, Bambo MP, Pinilla J, Larrosa JM, Satue M (2014). Reliability and validity of Cirrus and Spectralis optical coherence tomography for detecting retinal atrophy in Alzheimer’s disease. Eye.

[13] Kromer R, Serbecic N, Hausner L, Froelich L, Aboul-Enein F, Beutelspacher SC (2014). Detection of retinal nerve fiber layer defects in Alzheimer’s disease using SD-OCT. Front Psychiatry.

[14] Shi Z, Wu Y, Wang M, Cao J, Feng W, Cheng Y (2014). Greater attenuation of retinal nerve fiber layer thickness in Alzheimer’s disease patients. J Alzheimers Dis.

[15] Shen Y, Shi Z, Jia R, Zhu Y, Cheng Y, Feng W (2013). The attenuation of retinal nerve fiber layer thickness and cognitive deterioration. Front Cell Neurosci.

[16] Hinton DR, Sadun AA, Blanks JC, Miller CA (1986). Optic-nerve degeneration in Alzheimer’s disease. N Engl J Med.

[17] Curcio CA, Drucker DN (1993). Retinal ganglion cells in Alzheimer’s disease and aging. Ann Neurol.

[18] Davies DC, McCoubrie P, McDonald B, Jobst KA (1995). Myelinated axon number in the optic nerve is unaffected by Alzheimer’s disease. Br J Ophthalmol.

[19] McKhann G, Drachman D, Folstein M, Katzman R, Price D, Stadlan EM (1984). Clinical diagnosis of Alzheimer’s disease: report of the NINCDS-ADRDA Work Group under the auspices of Department of Health and Human Services Task Force on Alzheimer's Disease. Neurology.

[20] American Psychiatric Association (1994). Diagnostic and Statistical Manual of Mental Disorders (DSM-IV).

[21] Petersen RC, Smith GE, Waring SC, Ivnik RJ, Tangalos EG, Kokmen E (1999). Mild cognitive impairment: clinical characterization and outcome. Arch Neurol.

[22] Berisha F, Feke GT, Trempe CL, McMeel JW, Schepens CL (2007). Retinal abnormalities in early Alzheimer’s disease. Invest Ophthalmol Vis Sci.

[23] Lu Y, Li Z, Zhang X, Ming B, Jia J, Wang R (2010). Retinal nerve fiber layer structure abnormalities in early Alzheimer’s disease: evidence in optical coherence tomography. Neurosci Lett.

[24] Paquet C, Boissonnot M, Roger F, Dighiero P, Gil R, Hugon J (2007). Abnormal retinal thickness in patients with mild cognitive impairment and Alzheimer’s disease. Neurosci Lett.

[25] Parisi V, Restuccia R, Fattapposta F, Mina C, Bucci MG, Pierelli F (2001). Morphological and functional retinal impairment in Alzheimer’s disease patients. Clin Neurophysiol.

[26] Armstrong RA (1996). Visual field defects in Alzheimer’s disease patients may reflect differential pathology in the primary visual cortex. Optom Vis Sci.

[27] Schlotterer G, Moscovitch M, Crapper-McLachlan D (1984). Visual processing deficits as assessed by spatial frequency contrast sensitivity and backward masking in normal ageing and Alzheimer’s disease. Brain.

[28] Cogan DG (1985). Visual disturbances with focal progressive dementing disease. Am J Ophthalmol.

[29] Nissen MJ, Corkin S, Buonanno FS, Growdon JH, Wray SH, Bauer J (1985). Spatial vision in Alzheimer’s disease. General findings and a case report. Arch Neurol.

[30] Kergoat H, Kergoat MJ, Justino L, Chertkow H, Robillard A, Bergman H (2001). An evaluation of the retinal nerve fiber layer thickness by scanning laser polarimetry in individuals with dementia of the Alzheimer type. Acta Ophthalmol Scand.

[31] Koronyo Y, Salumbides BC, Black KL, Koronyo-Hamaoui M (2012). Alzheimer’s disease in the retina: imaging retinal abeta plaques for early diagnosis and therapy assessment. Neurodegener Dis.

[32] Koronyo-Hamaoui M, Koronyo Y, Ljubimov AV, Miller CA, Ko MK, Black KL (2011). Identification of amyloid plaques in retinas from Alzheimer’s patients and noninvasive in vivo optical imaging of retinal plaques in a mouse model. NeuroImage.

[33] Williams PA, Thirgood RA, Oliphant H, Frizzati A, Littlewood E, Votruba M (2013). Retinal ganglion cell dendritic degeneration in a mouse model of Alzheimer’s disease. Neurobiol Aging.

[34] Guo L, Duggan J, Cordeiro MF (2010). Alzheimer’s disease and retinal neurodegeneration. Curr Alzheimer Res.

